# Genomic Characterization of a Coxsackievirus A20 Strain Recovered from a Child with Acute Flaccid Paralysis in Nigeria

**DOI:** 10.1128/MRA.00849-19

**Published:** 2019-10-17

**Authors:** T. O. C. Faleye, O. M. Adewumi, O. T. Olayinka, J. A. Adeniji

**Affiliations:** a Department of Virology, Faculty of Basic Medical Sciences, College of Medicine, University of Ibadan, Ibadan, Nigeria; b Center for Human Virology and Genomics, Department of Microbiology, Nigerian Institute for Medical Research, Lagos, Nigeria; c WHO National Polio Laboratory, University of Ibadan, Ibadan, Nigeria; Queens College

## Abstract

In light of recurrent outbreaks of circulating vaccine-derived poliovirus 2 (cVDPV2) in Nigeria, we describe the genome sequence of a coxsackievirus A20 strain (CVA20). The nonstructural region (NSR) of this CVA20 genome showed that the enigmatic NSRs in recombinant cVDPV2s (GenBank accession numbers JX275140 and KX162716) found in Nigeria were from indigenous enterovirus species Cs (EV-Cs).

## Abstract (precis)

In light of recurrent outbreaks of circulating vaccine-derived poliovirus 2 (cVDPV2) in Nigeria, we describe the genome sequence of a coxsackievirus A20 strain (CVA20).

## ANNOUNCEMENT

Enteroviruses (EVs) belong to the genus Enterovirus, family *Picornaviridae*, and order *Picornavirales*. The genus has 15 species, and poliovirus (the best characterized member of the genus) belongs to species C. Several coxsackie A viruses (e.g., CVA20) and some numbered EVs are nonpolio enterovirus members of species C (NPEV-C).

In 2016, we detected a CVA20 strain from a child with acute flaccid paralysis (AFP) in Nigeria (1). The virus produced cytopathic effect (CPE) on the initial inoculation of stool suspension from the child into the L20B cell line, but the CPE was not reproducible on passage into the L20B and RD cell lines. The agent causing the irreproducible CPE was typed as CVA20 using reverse transcription-PCR amplification of part of the VP1 gene and Sanger sequencing (1). In this study, the L20B supernatant was passaged into the HEp 2C cell line, incubated at 37°C, and observed for CPE daily using an inverted microscope. The CPE developed in the HEp 2C cell line after 7 days of incubation and was reproducible in the HEp 2C cell line. Viral RNA was extracted from the isolate using a total RNA extraction kit (Jena Bioscience, Jena, Germany), and complementary DNA (cDNA) was subsequently generated by using random hexamers with the Script cDNA synthesis kit (Jena Bioscience) as recommended by the manufacturer.

The genome of the isolate was amplified in overlapping fragments (2 to 3 kb each) as previously described (2). The amplicons generated were pooled and shipped to a commercial facility (MR, TX, USA), where library preparation and NextGen sequencing was done. Paired-end sequencing was performed (2 × 150 bp) for 300 cycles using the HiSeq system (Illumina). Assembly was completed using Kiki v0.0.9 and MEGAHIT v2.3.0 on the KBase platform (3) and the enterovirus genotyping tool (EGT) (4) with default settings. Analysis for similarity and recombination was done using SimPlot v3.5 (5) with default settings.

**FIG 1:**
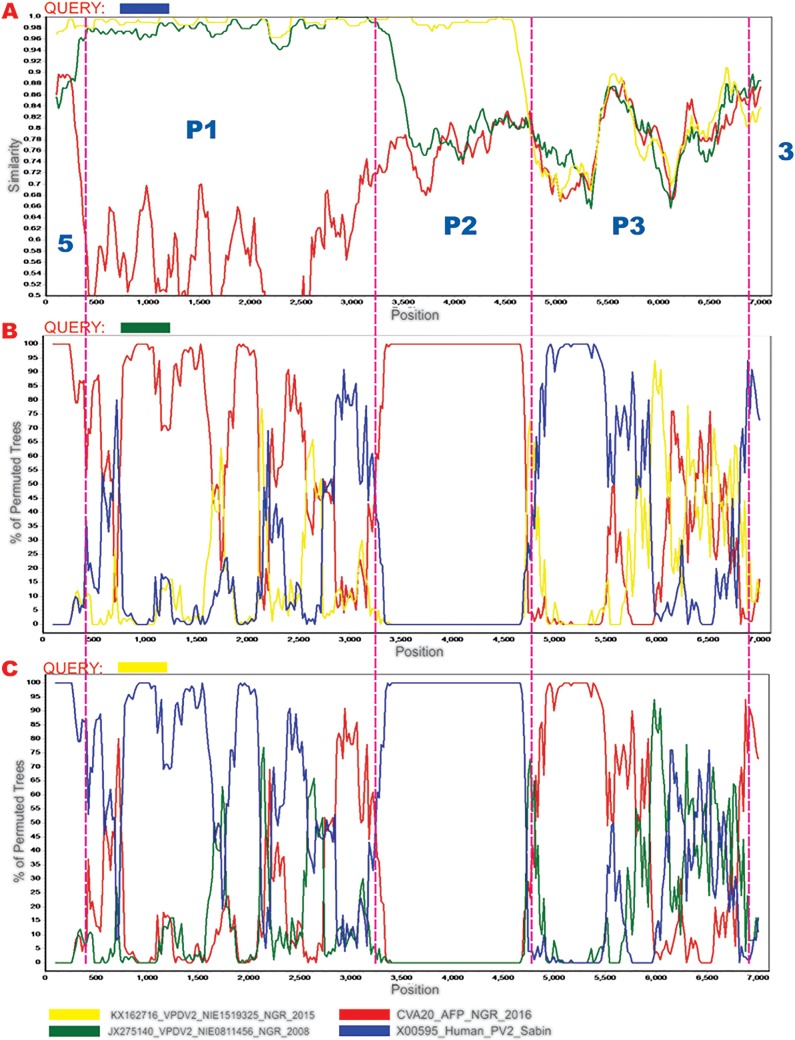
Similarity plot and bootscan analysis of the CVA20 strain described in this study with NIE0811456, NIE1519325, and Sabin 2 (GenBank accession number X00595) as the queries. The EV genome is a positive-sense RNA genome with untranslated regions (UTRs) on both ends (5′ and 3′). The large ORF is autocatalytically cleaved first into three smaller polypeptides (P1, P2, and P3) and then subsequently into 11 proteins. P1 encodes the capsid proteins, while P2 and P3 encode the nonstructural proteins. Hence, the designations 5 and 3 in panel A depict the UTRs at both ends of the genome. P1 depicts the capsid coding region, while P2 and P3 depict the nonstructural regions. (A) Similarity plot with the poliovirus 2 vaccine strain (Sabin 2) as the query sequence. It shows how similar the other three sequences are to the vaccine strain. (B) Bootscan analysis with the cVDPV2 strain recovered in Nigeria in 2008 (NIE0811456) as the query sequence. It shows how similar different regions of the other three genomes are to the query genome and provides evidence of recombination between the query genome and the others. (C) Same as for panel B but with the cVDPV2 strain recovered in Nigeria in 2015 (NIE1519325) as the query.

The genome for CVA20 strain NGR_2016 (GenBank accession number MH785183), with 7,091 nucleotides (nucleotides 289 to 7381 relative to the poliovirus reference sequence, GenBank accession number NC_002058), was assembled from 865,833 (24.4%) of the 3,548,364 reads generated. The genome has a G+C content of 45% and encodes two proteins (65 and 2,208 amino acids) in overlapping open reading frames (ORFs) (6). The isolate was confirmed as CVA20 by the EGT (4) (data not shown). Phylogenetic analysis and a BLAST search further confirmed the isolate as CVA20 and also showed that the P2 and P3 nonstructural regions of the virus were most similar to those of strains NIE0811456 (GenBank accession number JX275140) (87.09%) and NIE1519325 (GenBank accession number KX162716) (86.70%). NIE0811456 and NIE1519325 are recombinant circulating vaccine-derived poliovirus 2 strains (cVDPV2s) with a nonstructural region of unknown origin recovered in Nigeria in 2008 (7) and 2015 (8), respectively. SimPlot analysis confirmed that CVA20 strain NGR_2016 was most similar to strains NIE0811456 and NIE1519325 in the P2 and 5′ third of the P3 nonstructural region of the genome, respectively (Fig. 1). It also showed that the enigmatic 5′ untranslated region (UTR) of NIE0811456 (7) was very similar to that of NGR_2016 (Fig. 1B). Therefore, our findings show that NPEV-Cs circulating in the country were the source of a significant portion of the enigmatic regions of both recombinant cVDPV2s (NIE0811456 and NIE1519325).

### Data availability.

The genome described here has been deposited in GenBank under the accession number MH785183. The raw reads have also been deposited in the SRA with the BioProject accession number PRJNA554498.

## References

[1] Adeniji JA, Ibok UI, Ayinde OT, Oragwa AO, George UE, Faleye TOC, Adewumi MO 2018 Recovery of nonpolio enteroviruses from L20B cell line with non-reproducible cytopathic effect. J Adv Microbiol 9:2456–7116. doi:10.9734/JAMB/2018/40046.

[2] Faleye TOC, Adewumi OM, Adeniji JA 2018 Reference echovirus 7 and 19 genomes from Nigeria. Microbiol Resour Announc 7:e01465-18. doi:10.1128/MRA.01465-18. 30533861 PMC6284093

[3] Arkin AP, Cottingham RW, Henry CS, Harris NL, Stevens RL, Maslov S, Dehal P, Ware D, Perez F, Canon S, Sneddon MW, Henderson ML, Riehl WJ, Murphy-Olson D, Chan SY, Kamimura RT, Kumari S, Drake MM, Brettin TS, Glass EM, Chivian D, Gunter D, Weston DJ, Allen BH, Baumohl J, Best AA, Bowen B, Brenner SE, Bun CC, Chandonia JM, Chia JM, Colasanti R, Conrad N, Davis JJ, Davison BH, DeJongh M, Devoid S, Dietrich E, Dubchak I, Edirisinghe JN, Fang G, Faria JP, Frybarger PM, Gerlach W, Gerstein M, Greiner A, Gurtowski J, Haun HL, He F, Jain R, Joachimiak MP, Keegan KP, Kondo S, Kumar V, Land ML, Meyer F, Mills M, Novichkov PS, Oh T, Olsen GJ, Olson R, Parrello B, Pasternak S, Pearson E, Poon SS, Price GA, Ramakrishnan S, Ranjan P, Ronald PC, Schatz MC, Seaver SMD, Shukla M, Sutormin RA, Syed MH, Thomason J, Tintle NL, Wang D, Xia F, Yoo H, Yoo S, Yu D 2018 KBase: the United States Department of Energy Systems Biology Knowledgebase. Nat Biotechnol 36:566–569. doi:10.1038/nbt.4163. 29979655 PMC6870991

[4] Kroneman A, Vennema H, Deforche K, vd Avoort H, Peñaranda S, Oberste MS, Vinjé J, Koopmans M 2011 An automated genotyping tool for enteroviruses and noroviruses. J Clin Virol 51:121–125. doi:10.1016/j.jcv.2011.03.006. 21514213

[5] Lole KS, Bollinger RC, Paranjape RS, Gadkari D, Kulkarni SS, Novak NG, Ingersoll R, Sheppard HW, Ray SC 1999 Full length human immunodeficiency virus type 1 genomes from subtype C infected seroconverters in India, with evidence of intersubtype recombination. J Virol 73:152–160. 9847317 10.1128/jvi.73.1.152-160.1999 PMC103818

[6] Lulla V, Dinan AM, Hosmillo M, Chaudhry Y, Sherry L, Irigoyen N, Nayak KM, Stonehouse NJ, Zilbauer M, Goodfellow I, Firth AE 2018 An upstream protein-coding region in enteroviruses modulates virus infection in gut epithelial cells. Nat Microbiol 4:280–292. doi:10.1038/s41564-018-0297-1. 30478287 PMC6443042

[7] Burns CC, Shaw J, Jorba J, Bukbuk D, Adu F, Gumede N, Pate MA, Abanida EA, Gasasira A, Iber J, Chen Q, Vincent A, Chenoweth P, Henderson E, Wannemuehler K, Naeem A, Umami RN, Nishimura Y, Shimizu H, Baba M, Adeniji A, Williams AJ, Kilpatrick DR, Oberste MS, Wassilak SG, Tomori O, Pallansch MA, Kew O 2013 Multiple independent emergences of type 2 vaccine-derived polioviruses during a large outbreak in northern Nigeria. J Virol 87:4907–4922. doi:10.1128/JVI.02954-12. 23408630 PMC3624331

[8] Montmayeur AM, Ng TF, Schmidt A, Zhao K, Magaña L, Iber J, Castro CJ, Chen Q, Henderson E, Ramos E, Shaw J, Tatusov RL, Dybdahl-Sissoko N, Endegue-Zanga MC, Adeniji JA, Oberste MS, Burns CC 2017 High-throughput next-generation sequencing of polioviruses. J Clin Microbiol 55:606–615. doi:10.1128/JCM.02121-16. 27927929 PMC5277531

